# Perinatal testosterone exposure is critical for the development of the male-specific sexually dimorphic gastrin-releasing peptide system in the lumbosacral spinal cord that mediates erection and ejaculation

**DOI:** 10.1186/s13293-016-0058-x

**Published:** 2016-01-12

**Authors:** Takumi Oti, Keiko Takanami, Nao Katayama, Tomoca Edey, Keita Satoh, Tatsuya Sakamoto, Hirotaka Sakamoto

**Affiliations:** Ushimado Marine Institute (UMI), Graduate School of Natural Science and Technology, Okayama University, 130-17 Kashino, Ushimado, Setouchi, Okayama 701-4303 Japan

**Keywords:** Androgens, Sexual differentiation, Gastrin-releasing peptide, Male sexual function, Spinal cord

## Abstract

**Background:**

In rats, a sexually dimorphic spinal gastrin-releasing peptide (GRP) system in the lumbosacral spinal cord projects to spinal centers that control erection and ejaculation. This system controls the sexual function of adult males in an androgen-dependent manner. In the present study, we assessed the influence of androgen exposure on the spinal GRP system during a critical period of the development of sexual dimorphism.

**Methods:**

Immunohistochemistry was used to determine if the development of the spinal GRP system is regulated by the perinatal androgen surge. We first analyzed the responses of neonates administered with anti-androgen flutamide. To remove endogenous androgens, rats were castrated at birth. Further, neonatal females were administered androgens during a critical period to evaluate the development of the male-specific spinal GRP system.

**Results:**

Treatment of neonates with flutamide on postnatal days 0 and 1 attenuated the spinal GRP system during adulthood. Castrating male rats at birth resulted in a decrease in the number of GRP neurons and the intensity of neuronal GRP in the spinal cord during adulthood despite testosterone supplementation during puberty. This effect was prevented if the rats were treated with testosterone propionate immediately after castration. Moreover, treating female rats with androgens on the day of birth and the next day, masculinized the spinal GRP system during adulthood, which resembled the masculinized phenotype of adult males and induced a hypermasculine appearance.

**Conclusions:**

The perinatal androgen surge plays a key role in masculinization of the spinal GRP system that controls male sexual behavior. Further, the present study provides potentially new approaches to treat sexual disorders of males.

## Background

Early in life, androgens such as testosterone (T) and 5α-dihydrotestosterone (DHT) play a key role in the sexual differentiation of the central nervous system through a mechanism that is mediated by signaling through the nuclear androgen receptor (AR) [1–4]. In males, a considerable amount of T secreted transiently from the testes represents the “androgen surge” that masculinizes the developing brain as well as the external and internal genitalia [3–5]. Sex differences caused by the androgen surge are restricted to a limited period called the “sensitive” or “critical” period [4, 5]. For example, a critical period in rats occurs 18–27 days after fertilization when masculinization of the brain and spinal cord typically depends on transiently higher levels of circulating androgens [3, 4].

The spinal nucleus of the bulbocavernosus (SNB), which is located in the lumbosacral spinal cord (L5–S1 level), is homologous to Onuf’s nucleus in humans [6] and innervates the perineal-striated muscles attached to the base of the penis; the bulbocavernosus (BC) muscles in mammals such as rats [7, 8], mice [9], and monkeys [10]. The SNB-BC neuromuscular system is male-dominant in adults, and this sexual dimorphism is caused by sex differences in perinatal androgen exposure [7, 8]. Although androgens appear to play an important role in the establishment of SNB motoneuron number [11–14], somal size [15, 16], and neuromuscular synapse elimination [17], estrogens might also be critically involved in SNB dendritic development [18]. Taken together, these results suggest that, in the developing SNB-BC neuromuscular system, somal and dendritic growth may occur through separate developmental mechanisms, and that androgens and estrogens act synergistically to support normal masculine SNB dendritic development [8, 18].

Gastrin-releasing peptide (GRP), a brain-gut peptide, is expressed and distributed widely in the central nervous system as well as in the gastrointestinal tract of mammals [19]. GRP regulates many physiological processes, including food intake [20], circadian rhythms [21], anxiety [22], and the sensation of itch [23, 24]. Further, we demonstrated that neurons that express GRP (GRP neurons) in the lumbosacral spinal cord mediate the function of spinal centers that promotes penile reflexes during the sexual behavior of rats [25, 26]. Further, the number of GRP neurons in the lumbar spinal region (L3–L4 level) is greater in males than that in females [25, 26]. These GRP neurons project axons into the more caudal lumbosacral spinal cord (L5–S1 level), forming synaptic contacts with neurons in the sacral parasympathetic nucleus (SPN) and motor neurons in the SNB [25–30]. These nuclei control erection and ejaculation in an androgen-dependent manner during adulthood [26, 31, 32]. However, little is known about the effects of the androgen surge during perinatal development of this sexually dimorphic spinal GRP system.

To study the mechanisms underlying the development of the sexually dimorphic spinal GRP system in rats, we first determined the effects of administering the anti-androgen flutamide to neonatal male rats. Further, we castrated newborn male rats to prevent the production of endogenous androgens. In addition, we administered androgens to neonatal female rats to evaluate their effect on the development of the male-specific spinal GRP system during a critical period.

## Methods

### Animals

Male and female Wistar rats (Charles River Japan, Yokohama, Japan) were housed under a 12-h light/dark cycle and were provided unlimited access to water and rodent chow. The Committee for Animal Research, Okayama University, Japan authorized the experimental procedures.

### Flutamide treatment of male neonates

To determine if the development of the sexually dimorphic spinal GRP system is regulated by the androgen surge, flutamide [33, 34] (Sigma-Aldrich, St. Louis, MO, USA) or sesame oil (Nacalai, Kyoto, Japan) (control) was administered to male neonates under deep, cool anesthesia. Male pups from multiple dams were randomly divided into the groups as follows: male pups were injected subcutaneously (s.c.) with 0.25 mg flutamide in 100 μl of sesame oil on the day of birth (PND 0) and 24 h later (PND 1) (designated the demasculinization group, *n* = 5). This treatment resulted in a demasculinization of behavior in adult rats [33, 34]. Male pups (*n* = 5) from the same cohort were injected s.c. with 100 μl of sesame oil (designated the control group).

### Orchiectomy (ORX) and testosterone propionate (TP) treatment of male neonates

To examine the effects of the androgen surge on GRP expression in the lumbosacral spinal cord, neonates under deep, cool anesthesia were castrated and administered TP or sesame oil. Male pups from multiple dams were randomly divided into three groups. On PND 0, males (*n* = 3) underwent bilateral ORX to ablate androgen production and were then injected s.c. with 1.0 mg of TP (Nacalai) in 100 μl sesame oil and again on PND 1 for the purpose of androgen replacement (masculinization group) [35, 36]. Other males (*n* = 4) underwent bilateral ORX on PND 0 and were injected s.c. with 100 μl of sesame oil. Control males (*n* = 6) were sham-operated and injected s.c. with 100 μl of sesame oil on PNDs 0 and 1. On PND 30, which is approximately the onset of puberty, ORX or ORX + TP males were deeply anesthetized with 2 % isoflurane and implanted s.c. with 50-mm Silastic capsules (inner diameter = 1.59 mm, outer diameter = 3.18 mm; Compagnie de Saint-Gobain, Courbevoie, France) containing crystalline T (Tokyo Chemical, Tokyo, Japan) to maintain T levels similar to those of males at puberty for 50 days [26, 37]. Intact control males were implanted s.c. with empty 50-mm Silastic capsules.

### Ovariectomy (OVX) and TP or DHT treatments of female neonates

To assess the effect of androgen administration on the development of the male-specific spinal GRP system during a critical period in females, neonatal females under deep, cool anesthesia were administered androgens or sesame oil. Female pups from multiple dams were randomly assigned to five treatment groups. On PNDs 0 and 1, females (*n* = 5) were injected s.c. with 0.1 or 1.0 mg of TP in 100 μl of sesame oil, and other females (*n* ≥ 3) were injected s.c. with 0.1 or 1.0 mg with the non-aromatizable androgen DHT (Sigma-Aldrich) in 100 μl of sesame oil on PNDs 0 and 1 (all groups were designated masculinization groups). These doses of TP and DHT approximate the physiological levels present in female neonates [35, 38, 39]. Females (*n* = 6) from the same cohort were injected with 100 μl of sesame oil on PNDs 0 and 1 and served as controls. On PND 30, all females under deep isoflurane anesthesia underwent bilateral OVX to remove circulating estrogens, and 50-mm Silastic capsules containing T were immediately implanted s.c. into their backs where they remained for 54 days to maintain the levels of T similar to those of the males described above.

### Tissue preparation

After receiving the treatments described above, 11–12-week-old male and female rats were administered an overdose of sodium pentobarbital (100 mg/kg body weight) and perfused through the left ventricle with 100 ml of physiological saline followed by 200 ml of 4 % paraformaldehyde in 0.1 M phosphate buffer (PB; pH 7.4). Their spinal cords were immediately removed and fixed in the fixative described above for 3 h at room temperature.

### Immunohistochemistry (IHC) and immunofluorescence

The fixed lumbrosacral spinal cords were cryoprotected by immersing them in 25 % sucrose in 0.1 M PB for 48 h at 4 °C, quickly frozen using powdered dry ice, and cut into 30-μm-thick cross or horizontal sections using a cryostat (CM3050 S, Leica, Nussloch, Germany). Endogenous peroxidase activity was eliminated by incubating the sections in absolute methanol containing 1 % H_2_O_2_ for 30 min and then rinsed three times with phosphate buffered saline (PBS; pH 7.4) for 5 min. This procedure was omitted for samples subjected to immunofluorescence analysis. After blocking nonspecific binding sites with 1 % normal goat serum and 1 % bovine serum albumin in PBS containing 0.3 % Triton X-100 for 1 h at room temperature, the sections were incubated with a primary rabbit antiserum against GRP (1:2000 dilution) (11081; AssayPro, St. Charles, MO, USA) [24]. The specificity of the anti-GRP serum for the detected GRP in the spinal cord was demonstrated previously [24]. Immunoreactive (ir) products were detected using a streptavidin-biotin kit (Nichirei, Tokyo, Japan) and diaminobenzidine (Dojindo, Kumamoto, Japan) [24, 26, 40].

To determine the sites of projection of GRP-ir axons, we performed immunofluorescence analysis of the expression of GRP and neuronal nitric oxide synthase (nNOS). The latter serves as a marker for neurons in the SPN. To detect nNOS, we used a mouse monoclonal antibody (A-11, 1:5000 dilution; Santa Cruz Biotechnology, Santa Cruz, CA, USA). Both primary antibodies were combined to probe the sections. Alexa Fluor 546-conjugated anti-mouse IgG (Molecular Probes, Eugene, OR, USA) and Alexa Fluor 488-conjugated anti-rabbit IgG (Molecular Probes) were used for detection (each diluted 1:1000). Immunostained sections were imaged using a confocal laser scanning microscope (FluoView 1000, Olympus, Tokyo, Japan).

### Morphological analysis

We first counted GRP neurons in the lumbosacral spinal cord. The immunofluorescence analysis of GRP expression (GRP-ir) in neurons of the anterior lumbar spinal cord (L3–L4 level) was performed as described above using horizontal sections (approximately 18–22 30-μm-thick sections per animal) [26, 30, 37]. Briefly, we counted the number of GRP-ir cell bodies at ×200 magnification in all sections and analyzed a 600-μm^2^ area localized to the midline at the center. We acquired 5–15 micrographs per section, the number of which depended on the distribution of the GRP-ir neurons. These digital micrographs were selected and processed using Adobe PhotoShop (Adobe Systems, San Jose, CA, USA) and printed at 300 dots per inch on a photographic paper. GRP neurons were identified by their following characteristics: densely immunostained, anatomical localization (mainly dorsal, dorsolateral, or both to the central canal in lamina X of the lumbar segments III–IV), relatively large cell bodies (diameters approximately 20–30 μm), and clear round nuclei (diameters approximately 10–15 μm). To avoid overestimating cell number, only GRP-ir neurons that contained a round, transected nucleus were counted. Because the mean diameter of the nuclei in the GRP neurons is much smaller than the 30-μm-thick sections, this analysis reduced the overestimation of the number of neurons. All micrographs were coded and evaluated without the knowledge of the experimental group designation, and the code was not broken until the analysis was complete.

**Fig. 1:**
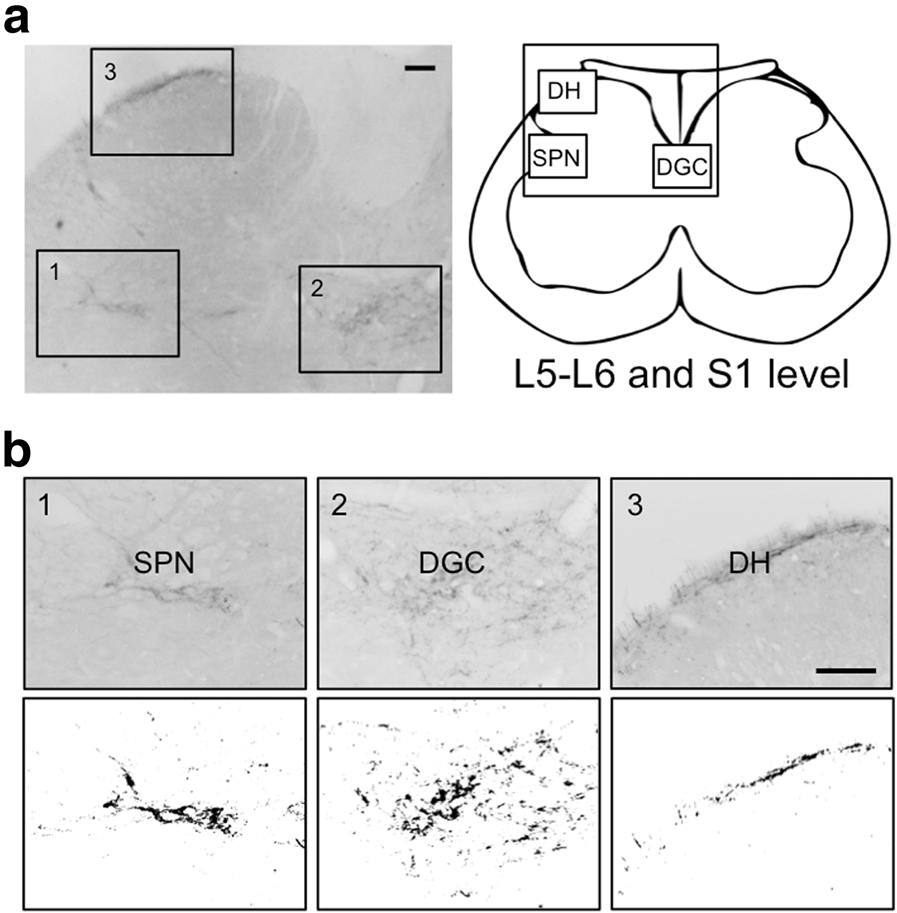
Semi-quantitative analysis of the expression of gastrin-releasing peptide (GRP) in the lumbosacral spinal cord (L5–S1 level). **a** Depiction of the lower lumbar and upper sacral spinal cord (L5–S1 level), indicating the intraspinal location of the sacral parasympathetic nucleus (SPN, a1), dorsal gray commissure (DGC, a2), and dorsal horn (DH, a3). The areas enclosed in boxes are enlarged in **b**. These magnified images show the distribution of GRP-immunoreactive fibers in the SPN (b1), DGC (b2), and DH (b3). The lower panels in **b** show the black-and-white images with a set threshold level corresponding to each upper panel. Scale bars 100 μm

We next performed a semi-quantitative analysis of GRP expression. To determine the density of GRP-ir fibers in the lumbosacral spinal cord (L5–S1 level), at least 10 cross sections (30-μm thick) per animal were randomly selected, and the digital images of three regions [the SPN, dorsal gray commissure (DGC), and dorsal horn (DH)] were prepared (magnification, ×200 per section). The unit area (343 × 469 μm^2^) was analyzed to localize the nuclei at the center of each area. The optical density of GRP staining was determined using black-and-white images that were converted from micrographs using ImageJ software (ImageJ 1.44p; National Institutes of Health, Bethesda, MD) (see Fig. 1) according to our established methods [26, 30, 40]. Briefly, the optical density of the background labeling was estimated by comparisons with similar areas of the control sections reacted with the anti-GRP antiserum that was incubated first with an excess of peptide antigen (50 μg/ml). GRP expression was undetectable in these sections. Each threshold, optical density was determined by normalizing the data to those of the preabsorbed sections. The GRP-ir-fiber pixel density was semi-quantitated as the average pixel density in the SPN, DGC, and DH of each animal, and the data were expressed as the ratio of each to the density of the DH, which was used as the standard in each analysis. Previous studies demonstrate that this procedure eliminates the variability in background staining among sections, animals, or both [26, 30, 40]. Moreover, this analysis substantially reflects mirrored changes in the levels of GRP mRNA and protein after androgen treatment of adult rats as we previously reported [37]. Micrographs were coded and evaluated without the knowledge of the experimental group designation, and the code was not broken until the analysis was complete.

### Enzyme immunoassay

Before perfusing the rats with formaldehyde, circulating blood was collected from the cardiac left ventricle, and the plasma was stored at −80 °C. To measure the T concentration, each plasma sample was assayed using a T enzyme immunoassay kit (Cayman Chemical, Ann Arbor, MI, USA) [37].

### Statistics

The number of GRP-ir neurons in the lumbar spinal cord of each group of animals was presented as the mean ± standard error of the mean (s.e.m.). The sex differences between males treated with flutamide and controls were assessed using the Student’s *t* test (mean ± s.e.m.). Statistical analyses of the number of GRP-ir neurons, the optical density of GRP-ir fibers, and plasma T concentrations (mean ± s.e.m.) were performed using one-way analysis of variance (ANOVA). When the significant main effects were found using ANOVA, the post hoc Tukey’s test or the Steel-Dwass test was performed.

## Results

### Administration of flutamide demasculinizes the spinal GRP system of neonatal male rats

**Fig. 2:**
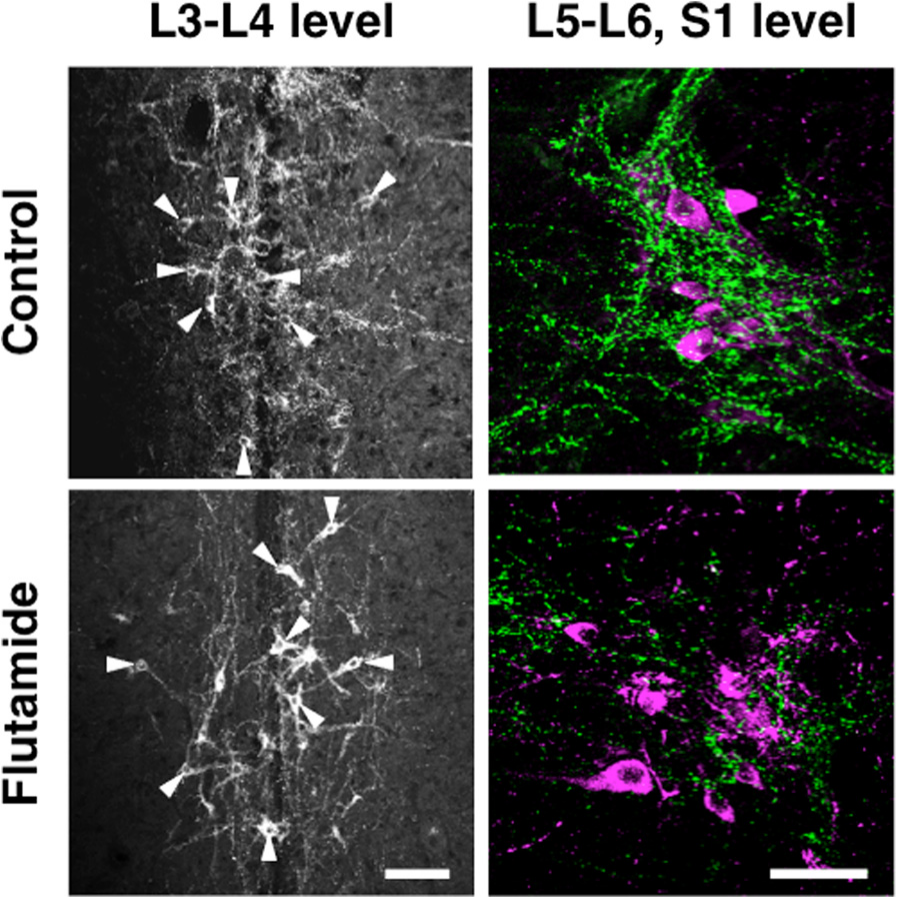
Flutamide administered to neonatal male rats demasculinizes the spinal GRP system. Flutamide decreased the number and intensity of GRP-immunoreactive neurons (*left panels*). Immunohistochemical analysis of the expression of GRP (*green*) and neuronal nitric oxide synthase (*magenta*) show a decrease in the intensity of GRP-immunoreactive fibers in the SPN. *Arrowheads* indicate possible GRP-positive cell bodies. Scale bars 100 μm, *left panel*; 50 μm, *right panel*

**Fig. 3:**
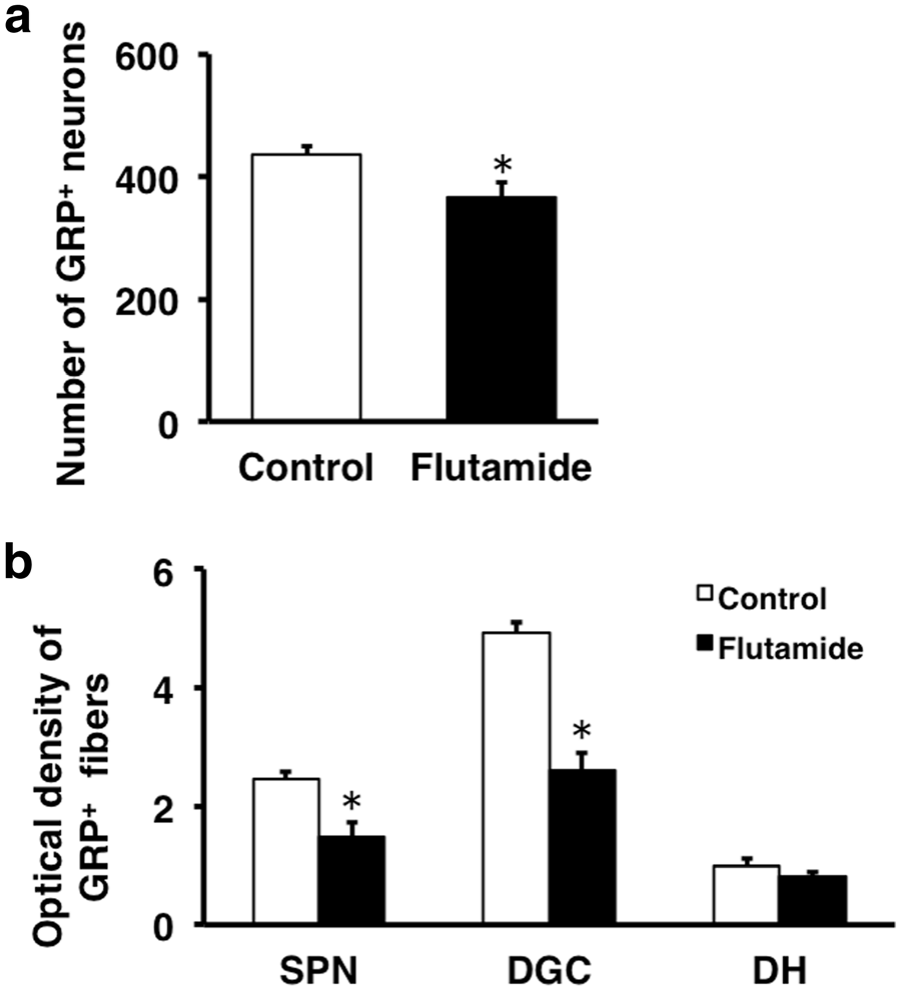
Semi-quantitative analyses of the effects on the spinal GRP system of the administration of flutamide to neonates. The number of GRP neurons (**a**) and the intensity of GRP-immunoreactive fibers (**b**) in the lumbosacral spinal cord. ^*^ *P* < 0.05 vs. control

To determine if the development of the sexually dimorphic spinal GRP system was regulated by the androgen surge, we administered the specific AR antagonist flutamide to neonatal male rats. Immunohistochemical analysis of GRP expression in the upper lumbar spinal cord (L3–L4 level) revealed slightly fewer GRP-ir neurons in neonatal flutamide-treated males than in controls (Fig. 2, left panels). Further, flutamide treatment decreased the intensity of GRP-ir dendrites in adult males (Fig. 2, left panels). Immunofluorescence analysis of GRP and nNOS expression revealed that the intensity of GRP-ir fibers was greater in control males than that in flutamide-treated males in the SPN, although the intensity of nNOS-ir staining was unchanged in the lumbosacral spinal cord (L5–S1 level) (Fig. 2, right panels). The number of GRP-positive neurons in the upper lumbar spinal cord (L3–L4 level) was significantly fewer in flutamide-treated males than that in control males [*t*(8) = 2.480, *P < 0.05] (Fig. 3a). The intensity of GRP-ir fibers in the lumbosacral spinal cord (L5–S1 level) was lower in flutamide-treated males than that in control males in the SPN [*t*(8) = 2.832, *P < 0.05] and DGC [*t*(8) = 5.718, *P < 0.05], but not in the DH [*t*(8) = 1.173] (Fig. 3b). In adults, the plasma concentrations of T were 4.34 ± 0.56 and 6.18 ± 1.25 ng/ml in oil-treated controls (*n* = 5) and the neonatal flutamide-treated group (*n* = 5), respectively. The adult level of plasma T after neonatal flutamide administration was not significantly different, which is consistent with the findings of a previous study [41].

### Effects of the androgen surge on neonatal development of the spinal GRP system in males

**Fig. 4:**
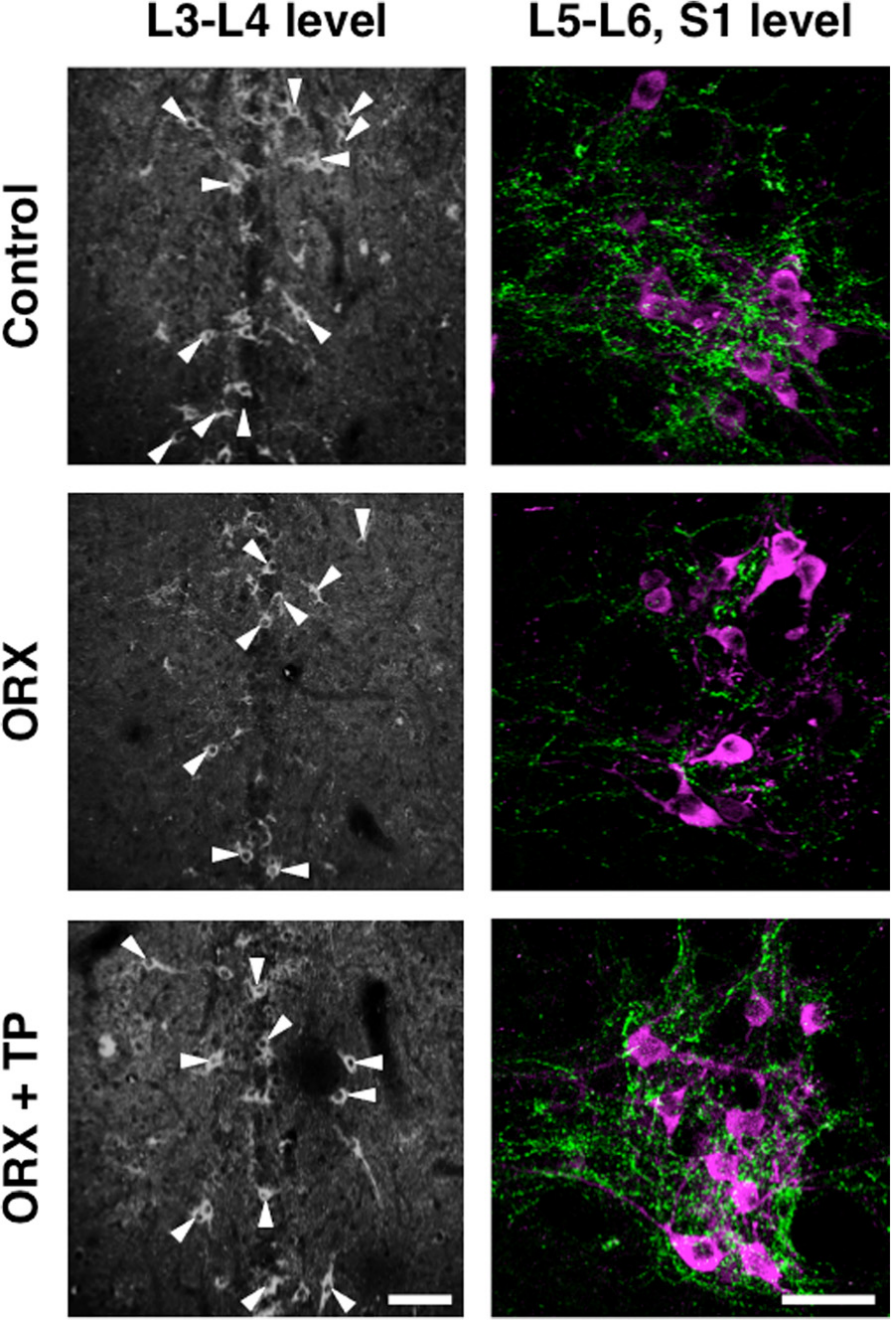
Neonatal castration demasculinizes the spinal GRP system. Effects of the androgen surge on neonatal development of the spinal GRP system in males (*left panels*). Immunohistochemical analysis of GRP (*green*) and nNOS (*magenta*) expression showed that the intensity of GRP-immunoreactive fibers in the SPN was decreased by neonatal orchiectomy (ORX) but was prevented by administering testosterone propionate (TP) (ORX + TP) to neonates. *Arrowheads* indicate possible GRP-positive cell bodies. Scale bars 100 μm, *left panel*; 50 μm, *right panel*

**Fig. 5:**
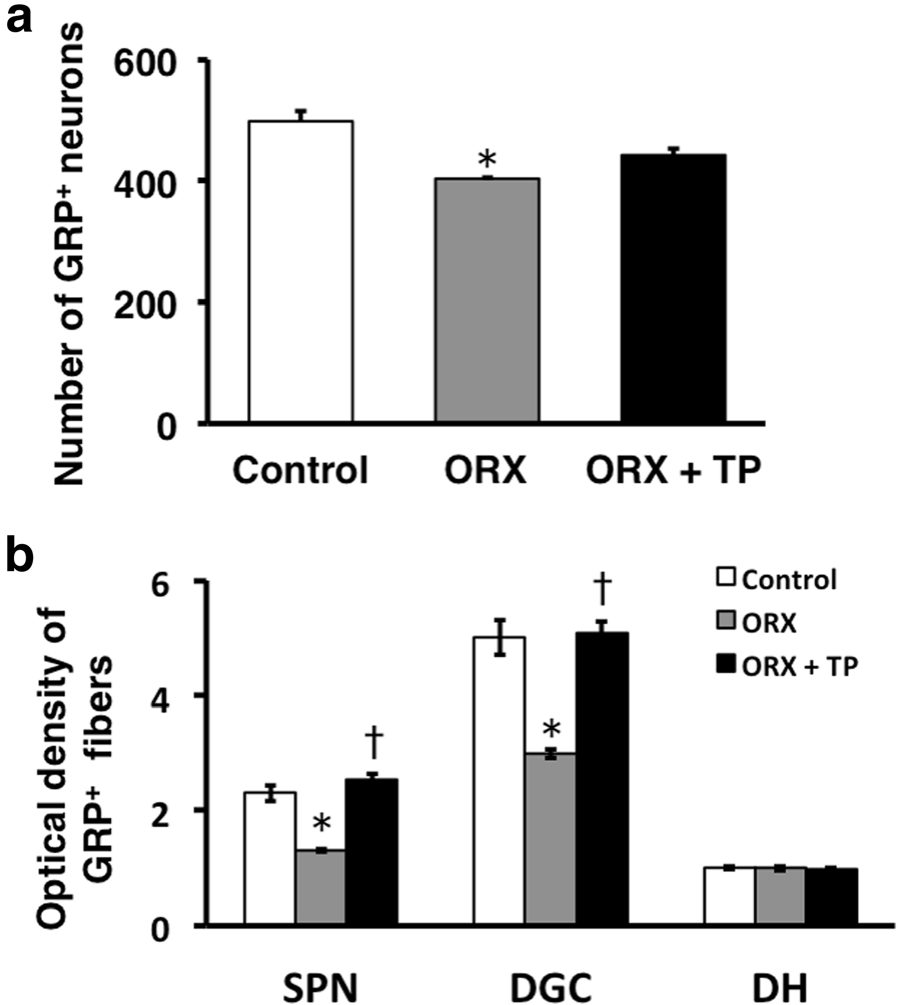
Semi-quantitative analysis of the effects of the androgen surge on the developing spinal GRP system of males. Castrating rats at birth (ORX) decreased the number and intensity of GRP-immunoreactive neurons in the spinal cord during adulthood. These effects were prevented if the castrates were treated with testosterone propionate (ORX + TP) immediately after castration. Number of GRP neurons (**a**) and intensity of GRP-immunoreactive fibers (**b**) in the lumbosacral spinal cord. ^*^ *P* < 0.01 vs. control; ^†^ *P* < 0.01 vs. ORX

We removed the testes of the neonates to assess the effects of the androgen surge on GRP expression in the lumbosacral spinal cord. ORX males were treated with T starting at PND 30. Immunohistochemical analysis of GRP expression in the upper lumbar spinal cord (L3–L4 level) of adults revealed fewer GRP-positive neurons and lower intensity of GRP-ir somata in ORX males than in control males, which were not significantly different in neonates (ORX + TP males) administered TP independent of T supplementation during adulthood (Fig. 4, left panels). Immunofluorescence analysis of GRP and nNOS expression in the SPN showed that the intensity of the GRP-ir fibers was lower in neonatal ORX males than in control and ORX + TP males (Fig. 4, right panels) independent of T supplementation during adulthood. The number of GRP-positive neurons in the upper lumbar spinal cord (L3–L4 level) was significantly decreased in ORX males compared with sham-operated controls [*F*(2, 10) = 12.37, *P < 0.01] (Fig. 5a). The intensity of GRP-ir fibers in the lumbosacral spinal cord (L5–S1 level) was greater in sham-operated controls than that in ORX males in the SPN [*F*(2, 10) = 24.91, *P < 0.01] and DGC [*F*(2, 10) = 18.67, *P < 0.01] but not in the DH [*F*(2, 10) = 0.109] (Fig. 5b). Because the number of GRP-positive neurons in ORX + TP-treated males was not significantly different compared with sham-operated male controls, neonatal T replacement restored the number of GRP-positive neurons in adults (Fig. 5a). Moreover, castrates displayed a significantly lower GRP expression than sham-operated groups and the ORX + TP-treated group in the SPN (^†^*P* < 0.01) and DGC (^†^*P* < 0.01), but the expression was constant in the DH (Fig. 5b). Because all castrates were implanted with T capsules on PND 30, plasma concentrations of T in sham-operated controls, ORX, and ORX + TP-treated males ranged from 2.16 ± 0.29 ng/ml (ORX and ORX + TP) to 3.45 ± 0.61 ng/ml (sham-operated control), suggesting that all the values were in the physiological ranges of those of the adult males.

### Neonatal administration of androgens masculinizes the spinal GRP system of females

**Fig. 6:**
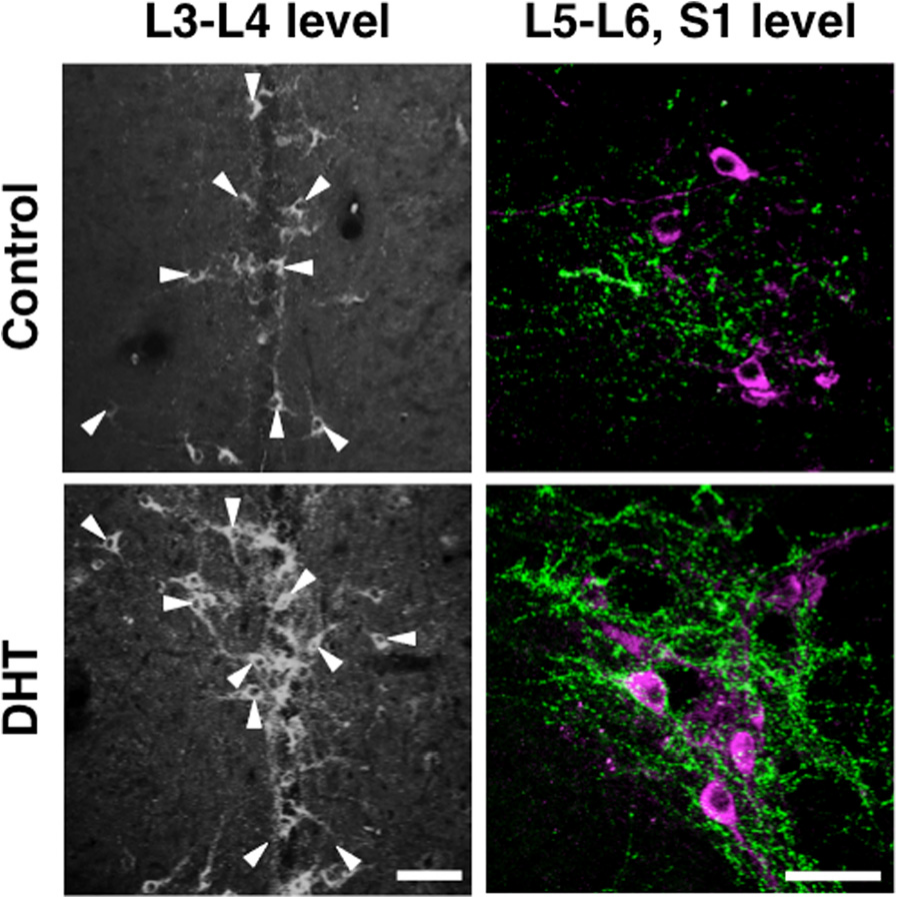
Masculinization of the spinal GRP system in female neonates exposed to androgens. Representative photomicrographs of female neonates treated with 5α-dihydrotestosterone (DHT). Neonatal administration of DHT increased the number and intensity of GRP-immunoreactive neurons (*left panels*). Immunohistochemical analysis of GRP (*green*) and nNOS (*magenta*) showing significantly increased intensity of GRP-immunoreactive fibers in the SPN induced by neonatal administration of DHT. *Arrowheads* indicate possible GRP-positive cell bodies. Scale bars 100 μm, *left panel*; 50 μm, *right panel*

**Fig. 7:**
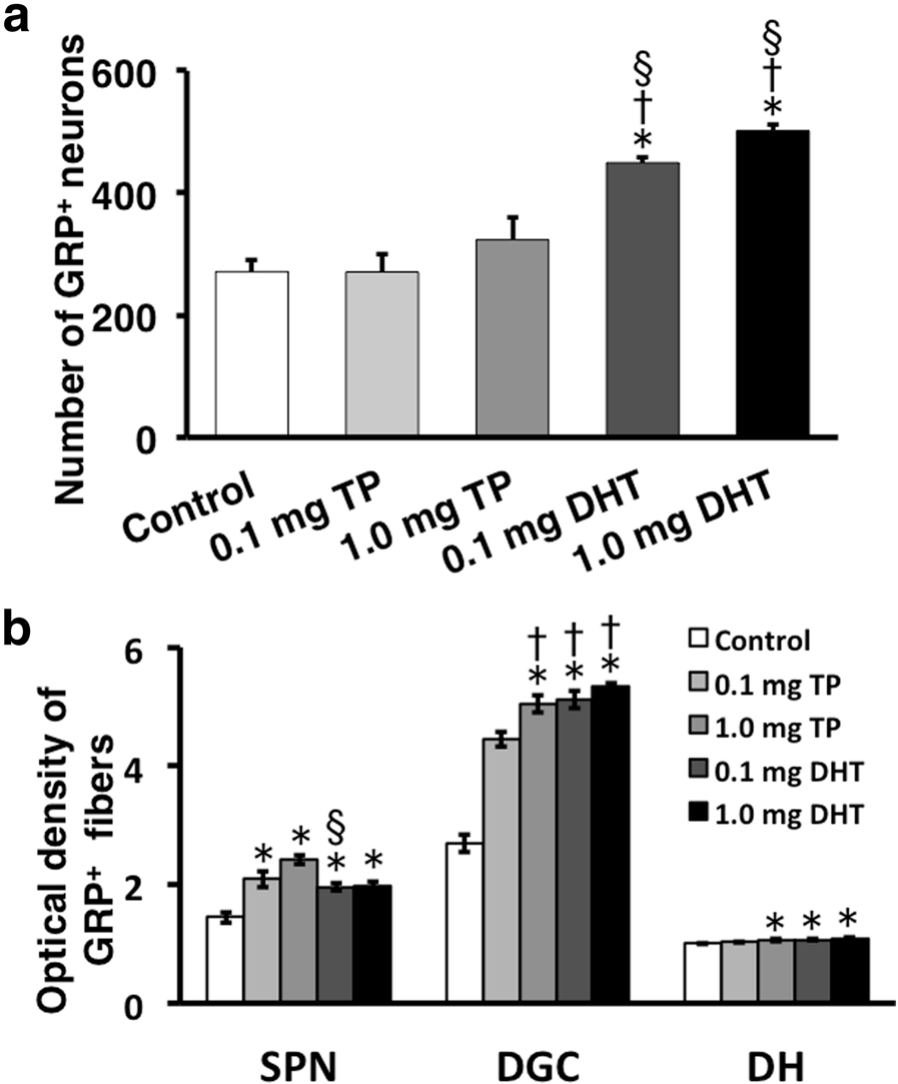
Semi-quantitative analyses of the effects of the androgen surge on the developing spinal GRP system in females. TP and DHT (0.1 or 1.0 mg each) were injected into female neonates on PNDs 0 and 1. The number of GRP neurons (**a**) and the intensity of GRP-immunoreactive fibers (**b**) in the lumbosacral spinal cord. ^*^ *P* < 0.05 vs. control; ^†^ *P* < 0.05 vs. 0.1 mg TP; ^§^ *P* < 0.05 vs. 1.0 mg TP

We administered androgens to females to assess the development of the male-specific spinal GRP system during a critical period (Fig. 6). Immunohistochemical analysis of GRP expression in the upper lumbar spinal cord (L3–L4 level) in neonatal females administered with DHT (1.0 mg) significantly increased the intensity of the GRP signal as well as the number of GRP-ir neurons compared with controls (Fig. 6, left panels). Immunofluorescence analysis of GRP and nNOS expression in the lumbosacral spinal cord (L5–S1 level) showed that the intensity of GRP-ir fibers in the SPN was greater in DHT-treated females than that in controls (Fig. 6, right panels). Moreover, there was no significant difference between the number of GRP neurons and the intensity of GRP-ir in the lumbosacral spinal cord of DHT-treated females compared with adult males (Fig. 6). To our knowledge, the present study is the first to show such an expression of male-specific GRP in the lumbosacral spinal cord of females. Moreover, the number of GRP-positive neurons in the upper lumbar spinal cord (L3–L4 level) was significantly greater in DHT-treated (0.1 and 1.0 mg) females than that in controls [*F*(4, 19) = 15.35, *P < 0.05] (Fig. 7a). Further, neonatal females administered with TP (0.1 and 1.0 mg) significantly increased the intensity of GRP-ir fibers in the SPN compared with controls [*F*(4, 19) = 15.68, *P < 0.05] and DGC [*F*(4, 19) = 66.60, *P < 0.05] (Fig. 7b), although there was no significant difference in the number of GRP-positive neurons (Fig. 7a). Further, the administration of DHT (0.1 and 1.0 mg) to neonatal females significantly increased the intensities of GRP-ir fibers relative to controls (*P < 0.05) in the SPN and DGC (Fig. 7b), and the expression levels were similar to those of adult males (Fig. 3b), mirroring the number of GRP cells (Fig. 7a, b). Significant increases in the levels of GRP-ir in the DGC were detected in the DHT-treated group (0.1 and 1.0 mg) and in the group treated with 1.0 mg of TP compared with the group treated with 0.1 mg of TP (^†^*P* < 0.05) (Fig. 7b). In the DH, small but significant differences in the intensity of GRP-ir fibers were observed among groups (*P < 0.05 vs. control) (Fig. 7b). All the females in this study were OVX and treated with T from PND 30. In adults, plasma T concentrations were slightly but statistically lower in 0.1 mg DHT-treated females than in oil-treated control only [*F*(4, 19) = 4.029, *P* < 0.05]. Nevertheless, plasma concentrations of T in oil-treated controls, TP-, and DHT-treated females ranged from 1.95 ± 0.15 (0.1 mg DHT) to 3.66 ± 0.44 ng/ml (oil-treated control), suggesting that all the values were in the physiological ranges of those of the adult males.

## Discussion

In the present study, we used rats to identify the mechanisms of sex-specific hormonal regulation, particularly androgen signaling, that mediate the development of the sexually dimorphic spinal GRP system. Here, we demonstrate that androgen treatment of neonates altered the spinal GRP system in adulthood. We were interested to find that neonatal androgen treatment induced complete masculinization of the spinal GRP system in XX females. To our knowledge, this is the first demonstration that the spinal cords of XX females were completely masculinized with respect to the spinal GRP system and that this male-specific sexual dimorphism persisted into adulthood.

### Androgens masculinize the spinal GRP system in XX females

In the present study, DHT treatment of female neonates increased the number and fiber density of GRP neurons in the lumbosacral spinal cord. This upregulation of the spinal GRP system in females approximated that of the adult males and led to a hypermasculine appearance. The intensities of GRP-fibers at the SPN and DGC were increased by treating neonates with TP, and the effect was dependent on the concentration of TP, although there were no significant changes in the number of GRP neurons in TP-treated groups. Thus, treating females with TP had no significant effect on the number of GRP-positive neurons, although TP increased the optical density of GRP-positive fibers. These findings indicate the possibility that administration of TP to neonates did not affect the number of GRP neurons but increased the level of ARs in the GRP neurons or altered androgen sensitivity. The higher levels of exogenous androgens, which are present in pubescent males, might increase the density of GRP-positive fibers during adulthood.

Together, these results indicate that DHT treatment of neonates induced masculinization of the spinal GRP system in contrast to the partial affect of TP. Because DHT binds with a higher affinity to the AR than the T, these differences in androgen actions suggest that the doses of TP used here were lower than the threshold level required to induce an effect in the developing spinal cord. Comparing these results with the SNB neuromuscular system in rats, the combination of prenatal and postnatal TP treatments can induce a complete masculinization of the SNB system in females [14, 39]. Goldstein and Sengelaub [38] found that the treatment of female perinatal rats with DHT propionate treatment increases the motoneuron number, somal size, and dendritic arborization in the SNB to the levels of an adult male. Our present results are consistent with these studies, suggesting that the androgen surge during perinatal life plays a significant role in masculinization of the spinal GRP system as well as that of the SNB-BC neuromuscular system. The synergistic effects of androgens on these spinal systems may play an important role in normal sexual differentiation in the rat spinal cord.

### Effects of androgens on sexual differentiation of the spinal GRP system during a critical period

Neonatal blockade of the androgen surge using the anti-androgen flutamide decreases the number and density of GRP neurons in the adult male spinal cord, although the endogenous T level is maintained or increased in adulthood [41, 42]. Moreover, we demonstrated that neonatal ORX decreased the number and density of GRP neurons in adulthood despite T supplementation administered around puberty (PND 30). Further, neonatal administration of TP to ORX pups, which might mimic the androgen surge, prevented the loss of the number or density of GRP neurons in the spinal cord during adulthood. We suggest therefore that the mechanisms involved in the neonatal effects of androgens on the spinal GRP system are related to the anti-apoptotic or neurotrophic effects of androgens in males during a critical period or are associated with the effects of epigenetic modifications.

We previously reported an entirely feminine pattern or a hyperfeminine appearance of the spinal GRP system in genetic males using two AR-deficient models as follows: (i) genetically male (XY) rats carrying a testicular feminization mutation of *AR* genes express a defective AR protein [26, 37] and (ii) a mouse line specifically lacking *AR* genes in the nervous system [40]. Nonetheless, analysis of these mutants did not address the involvement of AR in the development of the sexually dimorphic spinal GRP system during neonatal life because these two models lack AR function.

In contrast, a large body of literature shows that the androgen surge during a critical period also plays a pivotal role in the sexual differentiation of the rat brain [3, 4]. However, the masculinization of the brain appears to depend on estrogens converted from T by the enzymatic activities of the cytochrome P450 aromatase expressed in the brain [3, 4, 43]. The sexually dimorphic nucleus of the preoptic area (SDN-POA) is one of the most important in the brain that is involved in the regulation of male sexual behavior in rats [3, 4, 44]. Many studies have shown that the volume of the SDN-POA is several times larger in adult male rats than that in adult female rats [43–45]. Treating female rats with testosterone as well as a synthetic estrogen, diethylstilbestrol immediately before and after birth enlarges the size of the SDN-POA of adults to that of normal males [45], whereas the SDN-POA is smaller and feminized in adult male rats castrated at birth [3, 4]. Thus, it is suggested that estrogens are exclusively responsible for the establishment of this sexual dimorphism in the brain [3, 4, 43]. Similarly to the brain, it is likely that estrogens might play a role in a normal masculinization of the spinal GRP system. On the other hand, we show here that disrupting androgen signaling on PNDs 0 and 1 significantly attenuated the spinal GRP system, suggesting that these 2 days (PNDs 0 and 1) might be sufficient for masculinization of the spinal GRP system in rats. These findings also suggested that the incomplete demasculinization of the spinal GRP system in male rats may be attributed to the unpreventable effects of neonatal surgery or treatment on the androgen surge during embryonic life, the level of secreted T before castration on the day of birth, or both.

### Somatosensory GRP system in the spinal cord

Evidence indicates that the expression of GRP in the spinal DH is involved in the transmission of the itch sensation [23, 24]. We did not detect significant effects on the expression of GRP following neonatal administration of androgens or their antagonists on the expression of GRP in the adult spinal DH. Long-term ORX (28 days) or ORX combined with TP treatment of adult rats did not change the density of GRP-fibers in the DH of either sex [26, 37]. These results are consistent with present data, suggesting that androgens do not affect the expression of GRP in the spinal somatosensory system. These and the present findings show that the differences between the sexual and somatosensory systems in the spinal cord are not mediated by different modes of androgen signaling and that only one system is androgen sensitive.

## Conclusions

We demonstrated that the androgen surge during a critical period in rats plays a key role in the development of the sexually dimorphic GRP system in the lumbosacral spinal cord. Further understanding of the mechanisms of sexual differentiation in the spinal cord may provide new avenues for treating male sexual disorders.

## Abbreviations

AR: androgen receptor
BC: bulbocavernosus
DGC: dorsal gray commissure
DH: dorsal horn
DHT: 5α-dihydrotestosterone
GRP: gastrin-releasing peptide
IHC: immunohistochemistry
ir: immunoreactive
nNOS: neuronal nitric oxide synthase
ORX: orchiectomy
OVX: ovariectomy
PND: postnatal day
s.c.: subcutaneously
SDN-POA: sexually dimorphic nucleus of the preoptic area
SNB: spinal nucleus of the bulbocavernosus
SPN: sacral parasympathetic nucleus
T: testosterone
TP: testosterone propionate
